# Low-Intensity Shockwave Therapy in the Treatment of Erectile Dysfunction

**DOI:** 10.7759/cureus.11286

**Published:** 2020-11-01

**Authors:** Kelly Lurz, Paulette Dreher, Jason Levy, Brian McGreen, Javier Piraino, Andrew Brevik, Daniel Edwards, Laurence H Belkoff

**Affiliations:** 1 Urological Surgery, Mainline Health, Philadelphia, USA; 2 Urology, Mainline Health, Philadelphia, USA; 3 Urology, Kansas City University of Medicine and Biosciences, Kansas City, USA; 4 Surgery, Mainline Health, Philadelphia, USA

**Keywords:** erectile dysfunction, extracorporeal shockwave therapy, surveys and questionnaires

## Abstract

Introduction

Low-intensity shockwave therapy (LISWT) may improve erectile function in patients with mild to moderate erectile dysfunction (ED). Currently there is a paucity of research and prospective data on the utilization of LISWT in patients with ED. We present the results of our phase II clinical trial of LISWT with short-term follow-up in a cohort of patients with mild to moderate vasculogenic ED.

Methods

We obtained IRB approval and prospectively enrolled patients with mild to moderate vasculogenic ED. Baseline International Index of Erectile Function (IIEF) scores and peak systolic velocities (PSV) of cavernosal arteries measured on duplex penile ultrasound were obtained prior to treatment. Treatment included 6600 total shocks per session, for a total of six consecutive weekly treatment sessions. Baseline Erectile Dysfunction Inventory of Treatment Satisfaction (EDITS) scores were obtained at the completion of the treatment course. IIEF, EDITS and PSV were evaluated again at one-month follow-up. Clinical significance was defined as a median IIEF score increase of four points from baseline or an EDITS total score increase to greater than 65 or increase of greater than ten from baseline. Treatment success was evaluated on an individual basis and defined by a clinically significant improvement in questionnaire score.

Results

A total of 25 patients were enrolled in the trial, with 22 patients reporting for one-month follow-up. 68% (15/22) of patients demonstrated treatment success. In the cohort there was improvement in median EDITS from 61 (IQR 49-92) to 73 (IQR 43-49), which did meet criteria for clinical significance, but did not reach statistical significance (p = 0.74). IIEF improved from a median of 13 (IQR 12-19) to 18 (IQR 14-25), which did reach statistical significance (p = 0.011). On duplex ultrasound, mean cavernosal artery PSV increased from 34.3 cm/s (IQR 25.7-51.1) to 38.0 cm/s (IQR 31.6-45.1); however, these differences were statistically insignificant (p = 0.986). Of the 25 patients undergoing LISWT, two reported discomfort during treatment sessions, which subsided after repositioning the device without alterations in energy delivered.

Conclusion

LISWT may be a safe and potentially efficacious clinical modality for treatment of patients with mild to moderate vasculogenic ED demonstrating increases in cavernosal artery PSV and improvements in IIEF and EDITS scores in short-term follow-up. Longitudinal studies with increased power are needed to better evaluate the long-term efficacy and cost-efficiency of this therapy.

## Introduction

Erectile dysfunction (ED), described by the American Urological Association (AUA) as the inability to achieve and maintain an erection sufficient for satisfactory sexual intercourse, is estimated to affect upwards of 30 million men in the United States. Arising from both organic and psychogenic etiologies, over 600,000 American men are diagnosed with ED every year [1]. Of all organic ED etiologies, vasculogenic causes are the most extensive in the United States, likely related to the prevalence of obesity, cardiovascular disease, and diabetes in Western society [2]. The management of vasculogenic ED has historically been focused on treatment of the symptom, flaccidity, rather than long-term correction of the underlying mechanism attributed to vascular and endothelial dysfunction. However, studies have demonstrated that lifestyle modifications, such as diet, exercise, weight loss, and smoking cessation may contribute to long-term improvements in ED, likely due to improved endothelial function [3].

The clinical diagnosis of ED is most commonly made with the assistance of validated questionnaires; the International Index of Erectile Function (IIEF) and Erectile Dysfunction Inventory of Treatment Satisfaction (EDITS) are two of the most frequently used. Along with identifying the nature of ED, these questionnaires can be utilized to monitor symptom improvement and treatment outcomes. While oral phosphodiesterase-5-inhibitors (PDE5-Is), vacuum devices, intraurethral alprostadil, intracavernosal injections, and penile prosthesis surgery remain at the forefront of treatment for ED, research is surfacing regarding the implication of low-intensity shockwave therapy (LISWT) in the treatment of ED [4].

LISWT involves the application of linear shockwaves to target tissues, resulting in cellular microtrauma [4]. In turn, this microtrauma leads to the upregulation and release of angiogenic factors resulting in neovascularization of tissue [4]. LISWT has been utilized in many fields to stimulate tissue regrowth and regeneration, most notably in orthopedics for muscle, joint, and tendon recovery [5]. Clinical trials in human populations, although typically small and heterogeneous in population, have suggested improvements in erectile function after therapies employing LISWT to the corporal bodies [4,6-9].

The purpose of the current study was to evaluate the safety and short-term efficacy of LISWT in the treatment of mild to moderate ED and to consider potential factors associated with treatment success or failure in this population.

## Materials and methods

Internal Review Board (IRB) approval was obtained through the Western IRB to perform a non-randomized prospective phase II clinical trial.

Patient selection

Patients diagnosed with organic ED in a large urology group practice were screened for enrollment and referred to the central research clinic if baseline selection criteria were met. Research assistants then verified eligibility criteria and patients were enrolled in the study. Inclusion criteria included male patients between the ages of 35 to 75 years with a diagnosis of mild, mild to moderate, and moderate ED based on IIEF erectile function domain scores (22-25, 17-21, and 11-16, respectively), as well as a prior response to oral PDE-5I therapy, suggesting vasculogenic pathology and potentially reversible endothelial dysfunction [10]. Patients were limited to those reporting involvement in a heterosexual monogamous relationship with a minimum of three months duration and agreement from both partners to attempt intercourse a minimum of four times per month. Patients were excluded if they had a prior history of genitourinary malignancy, pelvic radiation therapy, implantable penile prosthesis, artificial urethral sphincter, uncorrected hypogonadism, substance abuse in the past three years or medical comorbidities including unstable angina, myocardial infarction in the last six months, congestive heart failure, or arrhythmias.

Study design

**Figure 1:**
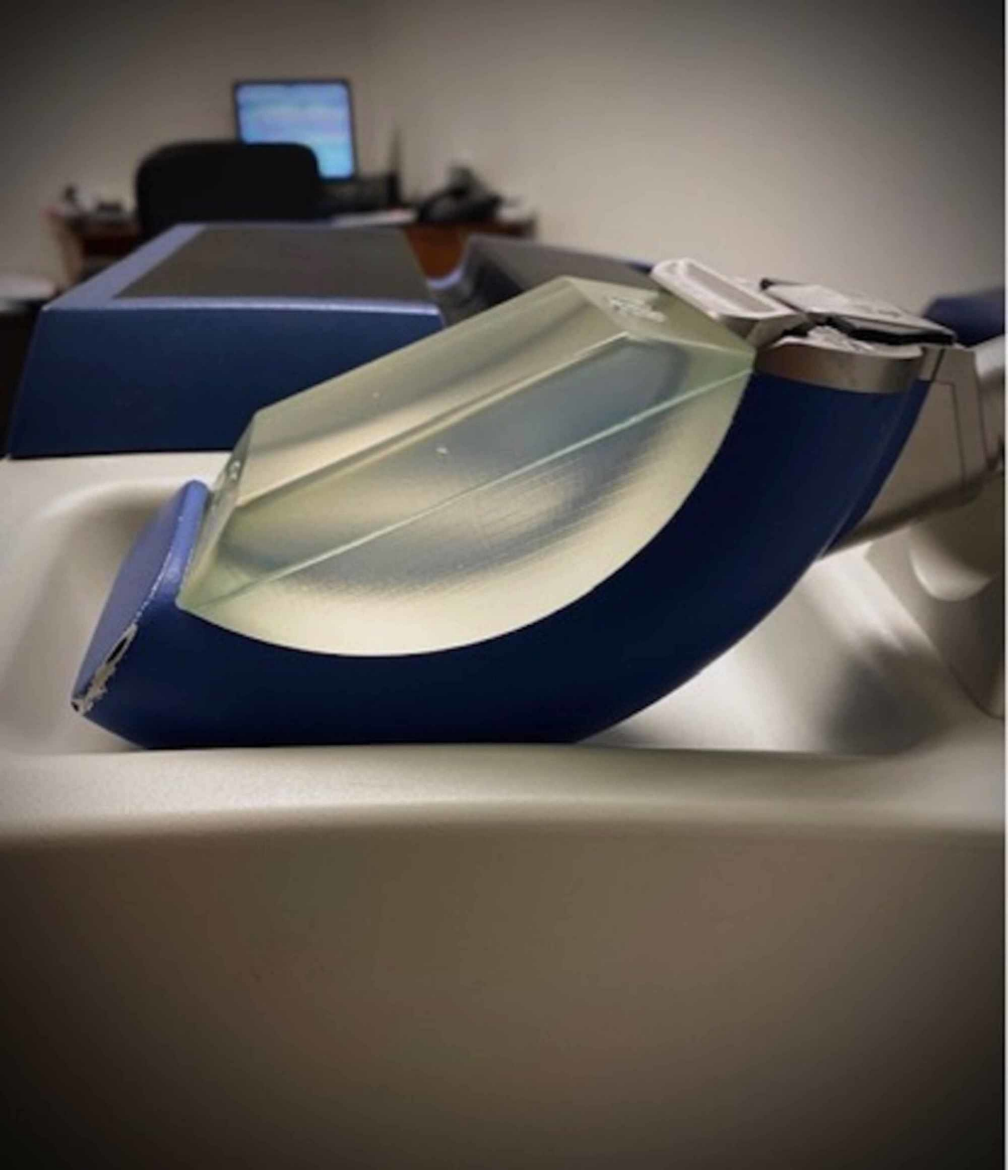
Richard-Wolf PiezoWave low intensity shockwave lithotripter

Subjective and objective outcomes were measured using pre- and post-treatment validated questionnaires and cavernosal artery velocities. Participants underwent a baseline screening evaluation including IIEF questionnaire and penile duplex ultrasound of the bilateral cavernosal arteries to determine peak systolic velocity (PSV). Patients then underwent treatment sessions once per week for six weeks using the Richard-Wolf PiezoWave low intensity shockwave lithotripter (Figure 1). Treatment sessions consisted of a total of 6,600 shocks (at 0.16 mJ/mm^2^), with 3,300 shocks to each of the bilateral corpora via the penile shaft and proximally to the crura via the perineum. At completion of the six-week course, patients were evaluated with an EDITS questionnaire, which is referred to as “baseline” throughout this study. Patients underwent subsequent evaluation utilizing IIEF and EDITS questionnaires and PSV at one month following the conclusion of their treatment course. Dedicated research personnel administered questionnaires and a designated ultrasonographer performed LISWT treatments as well as penile duplex ultrasounds. PSV value for each patient was calculated as the arithmetic mean peak velocity of the two cavernosal arteries.

Statistical analysis

​Statistical analysis was performed using IBM® SPSS® Statistics 24 (IBM Corp., Armonk, NY). Demographic and pre-treatment data were summarized by severity of baseline ED using medians and interquartile ranges for continuous variables and frequencies for categorical variables. Data were compared using Fisher’s exact test for categorical variables and either Wilcoxon sign rank or Kruskal-Wallis ANOVA for continuous variables, as appropriate.

Clinical significance was defined as a median IIEF score increase of four points from baseline, or an EDITS total score increase to greater than 65 or increase of greater than 10 from baseline at one-month follow-up. Clinically significant changes in IIEF scores utilized in the evaluation of LISWT for ED vary across the literature; therefore we derived and utilized a modified average of these values [11-12]. Clinically significant values for changes in EDITS were derived from data published by Cappelleri et al. that described a clinically important difference on EDITS as a score increase of 10 points [13]. Treatment success was evaluated on an individual basis and defined by a clinically significant improvement in questionnaire score. Changes in PSV were evaluated for statistical significance only. Statistical significance for all metrics was defined at a 95% confidence interval with p-value of <0.05.

## Results

Twenty-five patients completed all six weekly treatment sessions and 22 patients had follow-up at one month. Demographics and pre-treatment data were categorized by severity of baseline ED. Patients had a median age of 66 years (IQR 55-69) for mild ED and 60 years (IQR 56-67) for moderate ED. Median duration of ED was reported as 8.5 years for mild and 4.0 years for moderate ED (Table 1).

**Table 1:** Demographic data distribution by severity of baseline erectile dysfunction (ED).

		Mild (n = 4)	Mild to Moderate (n = 6)	Moderate (n = 15)	P-Value		
Age (years)		66 (55-69)	60 (56-67)	60 (55-61)	0.463		
BMI (kg/m^2^)		27.5 (24.9-33.8)	32.7 (24.4-37.6)	30.5 (27.9-33.5)	0.655		
Serum Testosterone (ng/dl)		333 (223-633)	404 (256-824)	571 (281-656)	0.765		
Duration of ED (years)		8.5 (3.0-14.8)	7.0 (1.5-18.5)	4.0 (3.0-7.3)	0.544		
Race					0.554		
	African-American	3	3	11			
	Caucasian	1	3	4			
Smoker					0.202		
	Yes	0	3	7			
	No	4	3	8			
Alcohol Use					0.736		
	Yes	3	5	10			
	No	1	1	5			
Hypertension					0.328		
	Yes	3	4	6			
	No	1	2	9			
Diabetes Mellitus					0.482		
	Yes	0	1	4			
	No	4	5	11			

On a per patient basis, 68% (15/22) experienced treatment success at one month post treatment. The calculated median EDITS score for all patients increased from 61 (IQR 49-92) to 73 (IQR 43-91) at one month, which is clinically, but not statistically significant (p = 0.744). IIEF-6 scores, however, demonstrated both statistical and clinical improvements, increasing from a median of 13 (IQR 12-19) to 18 (IQR 14-25) at one month (p = 0.011). Median EDITS and IIEF scores from baseline to one month follow-up for mild, mild to moderate, and moderate ED are displayed in Table 2 and Table 3, respectively.

**Table 2:** Changes in median EDITS scores from baseline to one-month post-treatment. Interquartile ranges listed in parentheses. EDITS: Erectile Dysfunction Inventory of Treatment Satisfaction

	Baseline	One Month	P-Value
Overall	61 (49-92)	73 (43-91)	0.744
Mild ED	75 (37-100)	89 (58-100)	0.317
Mild to Moderate ED	73 (57-87)	66 (53-90)	1.000
Moderate ED	63 (43-93)	64 (34-90)	0.889

**Table 3:** Changes in median IIEF scores from baseline to one-month post-treatment. Interquartile ranges listed in parentheses. IIEF: International Index of Erectile Function

	Baseline	One Month	P-Value
Overall	13 (12-19)	18 (14-25)	0.011
Mild ED	23 (22-25)	25 (25-25)	0.180
Mild to Moderate ED	18 (17-20)	23 (19-25)	0.102
Moderate ED	12 (11-13)	15 (10-23)	0.078

Cavernosal artery PSV increased from 34.3 cm/s (IQR 25.7-51.1) pre-treatment to a mean of 38.0 cm/s (IQR 31.6-45.1) at one-month post-treatment. Although the average PSV at one-month follow-up increased to greater than the defined lower limit of normal (35 cm/s), these changes were not statistically significant (p = 0.986) [14].

On univariate analysis, smoking status appeared to be associated with increased likelihood of treatment failure (OR 6.90, p = 0.074), but this failed to reach statistical significance. No other pretreatment characteristics appeared to be associated with success of treatment (p > 0.1).

Minor adverse events involving 1.3% (n = 2) of 150 treatment sessions necessitated altering shock delivery due to mild discomfort, but no sessions were terminated. Importantly, no adverse effects including penile hematoma, corporal rupture, or deep vein thrombosis were noted.

## Discussion

Shockwave therapy in the form of lithotripsy has long been used in the treatment of urolithiasis in order to directly break up stones at the time of treatment. However, shockwave therapy in the treatment of ED relies on delayed outcomes from tissue response to the damage created by shockwaves [15]. The hypothesized response mechanism is centered on local neovascularization. Wang et al. demonstrated substantially increased levels of endothelial nitric oxide synthase, vascular endothelial growth factor, and proliferating cell nuclear antigen when shockwave therapy was applied to Achilles tendons of rabbits [16]. Nerve regeneration and mesenchymal stem cell migration/proliferation has also been suggested as an outcome of shockwave therapy [15]. While these molecular-level changes have been demonstrated in vitro and in animal studies, the complete mechanism has not been fully understood in humans and research is ongoing.

Shockwave therapy has been employed in several other areas of medicine with promising results. After numerous porcine studies revealed LISWT improved regional myocardial blood flow and improved left ventricular remodeling after reperfusion injury, it was investigated in humans with ischemic heart disease [17-19]. In a small study by Kikuchi et al., promising results were reported in functional outcomes such as improved chest pain symptom scores and reduced nitroglycerin use, as well as objective outcomes like ejection fraction and stroke volume when cardiac LISWT was administered to men with severe ischemic disease [20]. A meta-analysis published in 2017 inclusive of 39 studies revealed consistent improvements in various clinical metrics such as quality of life and myocardial perfusion indices with the use of targeted LISWT in patients with stable coronary artery disease [21]. Shockwave therapy has been investigated in the treatment of diabetic foot ulcers with promising results when compared to hyperbaric oxygen therapy [22]. It has also been reported on extensively in the orthopedic literature, mainly in the treatment of over-use tendinopathies with success rates ranging from 65-91%. Shockwave therapy has even been FDA approved for the treatment of plantar fasciitis and lateral epicondylitis [5].

Several meta-analyses published within the past 10 years have found that LISWT can significantly improve patients’ symptoms as evaluated by validated questionnaires [7-9]. Lu et al. included 14 studies evaluating the efficacy of LISWT in patients with ED and reported statistically significant improvements in IIEF scores (95% CI, 0.99-3.00; p < 0.0001) and Erection Hardness Scores (EHS) (95% CI, 0.04-0.29; p = 0.01) [7]. A 2017 meta-analysis inclusive of 602 patients with ED who underwent treatment with either sham or LISWT demonstrated that LISWT had an average IIEF score increase of 6.40 (95% CI 1.78-11.02; P < 0.0001) compared to 1.65 (95% CI 0.92-2.39; P < 0.0001) compared to sham [8]. Vardi et al. reported improvements in IIEF scores from a mean of 13.5 at baseline to 20.9 one month after LISWT in 20 men with vasculogenic ED, congruent with the significant improvements seen in our IIEF scores from a baseline of 13 to 18 at one-month post-treatment [11]. Published data on LIWST is promising, but young and therefore the AUA considers it investigational. However, the European Association of Urology includes LIWST as a treatment option for patients with vasculogenic ED [23]. Our data supports the efficacy of LISWT as a treatment for mild to moderate ED, with 68% of patients experiencing improvement in symptoms and an average improvement in cavernosal artery PSV of approximately 4 cm/s at one-month follow-up.

The duration of treatment efficacy remains unclear. A meta-analysis from Man and Li evaluated 637 patients who received LISWT from years 2005-2017 for ED [9]. The authors utilized significant improvements in IIEF scores and EHS as their markers of treatment success; through this definition, therapeutic efficacy was noted up to three months post-treatment. Although our results do not extend past one-month follow-up, given the latter, perhaps peak improvements in erectile function were missed due to lack of longer-term data.

Shockwave devices vary in manufacturer, energy capacity, and shockwave delivery. Shockwaves may be delivered in a pinpoint manner, linear manner, or in a linear tissue-coverage manner. The device utilized in our study delivered shocks in a linear, tissue-coverage fashion. Our goal in utilizing a tissue-coverage approach was to deliver a more homogenous dose of energy with the hope that uniform microvascular changes would occur within the corpora bilaterally. In a study of 58 patients with mild to severe ED, Reisman et al. utilized a similar linear-tissue coverage device but with a curved applicator that partially wrapped around each corpora for maximal engagement with 81% reported success rate [12]. In a small study published in 2013, the use of a pinpoint shock applicator resulted in statistically significant improvements in IIEF scores and penile blood flow at one-month follow-up [24]. Despite promising data on individual shockwave applicators, there is limited data on device comparison and superiority.

It is rare to find consistent treatment protocols across trials utilizing LISWT for ED. Vardi et al. performed bi-weekly sessions for three weeks, repeated after a three-week treatment holiday [11]. They also required a four-week PDE-5I washout period prior to starting shockwave therapy. A study comparing efficacy of 6 versus 12 treatment sessions within a six-week period reported improved sexual performance in those undergoing 12 sessions [25]. Shock and energy delivery are also variable. In most studies, shocks delivered range from 600 to 5,000 with energy intensities of 0.009 to 0.16 mJ/mm [11-12,24]. Kalyvianakis et al.'s data suggests improved subjective and objective outcomes when utilizing 0.10 mJ/mm^2^ versus 0.05 mJ/mm^2^, but no difference in two versus three weekly treatment sessions for a total of 12 sessions [25]. A 2017 meta-analysis suggests that lower energy density (0.09 mJ/mm^2^, 95% CI, 0.87-7.42; P = .01), increased number of pulses (3000 pulses per treatment, 95% CI, 3.18-7.05, P < .0001), and a shorter total treatment course (<6 weeks, 95% CI, 0.54-6.93; P = .02) resulted in better therapeutic efficacy [9]. Contrastingly, Lu et al. found that variation in machine design, application technique and protocol did not impact their outcomes [7]. Ultimately, the variances in protocols and shockwave devices make comparison and standardized clinical application of this therapy a challenge.

In a 2013 meta-analysis, Cao et al. reported a significantly increased risk of ED in patients that were either former or current smokers with odds ratios of 1.29 (95% CI: 1.07 to 1.47) and 1.51 (95% CI: 1.34 to 1.71), respectively [26]. Smoking has been shown to decrease the bioavailability of nitric oxide leading to endothelial dysfunction by the proposed mechanism of free radical formation and aromatic compound accumulation [27]. Smoking status negatively impacted the success rates of LISWT defined as an increase of 2 and 5 points on IIEF scores for mild and moderate ED, respectively, in a study that included 15 men with mild to moderate vasculogenic ED [28]. In this study, patients with a smoking index of greater than 20 had a statistically significant higher likelihood of treatment failure compared to those with a smoking index of less than 20 (50% vs. 9%) [28]. Our data is supportive, also revealing that smoking is associated with treatment failure.

LISWT has been reported to be a safe treatment modality for patients with ED. In a meta-analysis published in 2019 that reviewed eight randomized controlled clinical trials investigating LISWT for ED, no adverse events were reported across all studies [29]. Some concern may be raised for increased risk of bleeding when treating patients on anticoagulation. A study by Kalyvianakis et al. that included 138 men on anticoagulation or antiplatelet therapy who underwent LISWT for ED reported no bleeding events or other adverse reactions [30]. In our cohort, anticoagulation/antiplatelet use was not elucidated, but the only adverse event reported was discomfort during treatment for two patients, which resolved with device repositioning and did not require termination of either session.

There are several limitations to our study, including the small cohort with limited demographics, short-term follow-up, and lack of a control arm. Even with short-term follow-up of only one month, three patients were lost which could significantly impact results in this small cohort. Although protocol was standardized, variability in ultrasound technician may impact patient outcomes and results. Additionally, duplex ultrasounds were individually reviewed by radiologists and there is likely inherent variation in PSV results and interpretation.

## Conclusions

LISWT may be a reliable treatment option in patients experiencing mild to moderate ED. The results of this study demonstrate short-term efficacy with overall improvement in subjective outcomes measured by IIEF and EDITS validated questionnaires as well as objectively with improvements seen in PSV of the cavernosal arteries. Data is limited due to lack of long-term results and small sample size. Future studies with larger cohorts and standardized protocols are needed to better delineate the long-term efficacy and feasibility of LISWT as a recommended treatment option for patients with mild to moderate ED.
